# Biodiversity time series are biased towards increasing species richness in changing environments

**DOI:** 10.1038/s41559-023-02078-w

**Published:** 2023-06-05

**Authors:** Lucie Kuczynski, Vicente J. Ontiveros, Helmut Hillebrand

**Affiliations:** 1 Plankton Ecology Lab, Institute for Chemistry and Biology of the Marine Environment, Carl von Ossietzky University Oldenburg, Wilhelmshaven, Germany; 2 Institute of Aquatic Ecology, University of Girona, Girona, Spain; 3 Department of Life Sciences, Ben-Gurion University of the Negev, Be’er Sheva, Israel; 4 Helmholtz Institute for Functional Marine Biodiversity at the University of Oldenburg (HIFMB), Oldenburg, Germany; 5 Alfred-Wegener-Institute, Helmholtz Centre for Polar and Marine Research, Bremerhaven, Germany

**Keywords:** Biodiversity, Climate-change ecology, Community ecology, Freshwater ecology

## Abstract

The discrepancy between global loss and local constant species richness has led to debates over data quality, systematic biases in monitoring programmes and the adequacy of species richness to capture changes in biodiversity. We show that, more fundamentally, null expectations of stable richness can be wrong, despite independent yet equal colonization and extinction. We analysed fish and bird time series and found an overall richness increase. This increase reflects a systematic bias towards an earlier detection of colonizations than extinctions. To understand how much this bias influences richness trends, we simulated time series using a neutral model controlling for equilibrium richness and temporal autocorrelation (that is, no trend expected). These simulated time series showed significant changes in richness, highlighting the effect of temporal autocorrelation on the expected baseline for species richness changes. The finite nature of time series, the long persistence of declining populations and the potential strong dispersal limitation probably lead to richness changes when changing conditions promote compositional turnover. Temporal analyses of richness should incorporate this bias by considering appropriate neutral baselines for richness changes. Absence of richness trends over time, as previously reported, can actually reflect a negative deviation from the positive biodiversity trend expected by default.

## Abstract (web summary)

There is great interest in describing biodiversity change through time, but such analyses present various technical challenges. Here, using datasets for fish and birds, the authors show that a bias towards colonization over extinction can result in an increasing species richness over time, especially in short time series, and argue that studies should account for this bias.

## Main

**Fig. 1:**
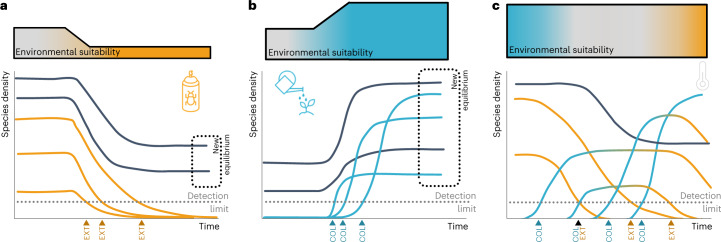
Conceptual figure of the impact of different anthropogenic changes on species diversity and species density. Yellow lines indicate species experiencing population declines up to extinction while blue ones indicate species experiencing increases in density. **a**, The case of a negative impact (for example, increase in pesticides, habitat fragmentation) resulting in a lower equilibrium richness, which can take some time to establish as declining populations persist (extinction debt). **b**, A clear positive impact (for example, enlargement of habitat size through restauration) that leads to a higher equilibrium richness, which might take time to establish as gained populations need some time to colonize (immigration credit). **c**, A steady change: even though as many species decline (that is, ‘losers’) as colonize (that is, ‘winners’), the observed richness increases if new species arrive earlier than species go extinct. This increase does not disappear, as any new time segment added leads again to earlier colonizations than extinctions, with no new equilibrium being reached. EXT, extinction; COL, colonization.

The expectation that species richness remains constant in the absence of external forcing at ecological time scales is deeply rooted in ecological theories^1,2^ assuming a dynamic equilibrium between colonizations and extinctions^3^. Assessments of time series in the global change context thus interpret deviations from balanced dynamics such as positive and negative trends in species number as a response to improving or deteriorating environmental conditions, respectively^4,5^. Under increased environmental suitability (Fig. 1), most species will profit, and the expected positive trends emerge, although colonizations may also be delayed (‘immigration credit’^6^). On the other hand, one can expect that a reduction in habitat suitability will affect most species negatively up to the extinctions of some (Fig. 1). As the exponential decline of existing populations takes time (for example, because of plasticity, use of microrefugia), extinction debts will lead to a delayed reduction in richness^6,7^ and the negative richness trends will only emerge later.

The scale- and effort-dependency of species richness as a metric creates uncertainty around trends^8,9^, while, in addition, richness does not capture compositional turnover but rather the net difference between colonizations and extinctions^10,11^. Even more fundamentally though, the temporal response of richness might not match our expectation, especially if the environment-driven trajectory is not clearly negative or positive (Fig. 1a,b) but neutral, as some species are favoured and can colonize while others decline and eventually go extinct (Fig. 1c).

To conceptualize the issue, consider a neutral environmental change such that there are equal numbers of ‘winners’ and ‘losers’, and richness is expected to remain constant. However, under low dispersal limitation, one can assume that colonizations (defined as the first colonization event over a given time series) will be fast (as it needs only few propagules), whereas extinctions (defined as the last extinction event over a given time series) will be delayed because in the absence of catastrophic mortality population growth will slowly turn negative for the losers. For dominant species, the resulting decline in abundance will result in extinction after many generations. This extinction process might be further slowed down if density-dependent mortality declines or populations adapt their phenotypes to the new conditions. This bias towards earlier colonizations will result in increasing richness over time, which may be transient if the environmental change stops at some point such that colonizations and extinctions can equilibrate again. However, if environmental change continues, each incremental increase in observation time will allow further colonizations, resulting in further imbalance detected as increasing richness in finite time series (Fig. 1c). On the other hand, if a community exhibits a strong inertia in its dynamics, rare species are likely to go extinct and not locally recolonize. Thus, as the majority of species are rare, decrease in richness will emerge.

## Results and discussion

### Temporal trends in species richness

**Fig. 2:**
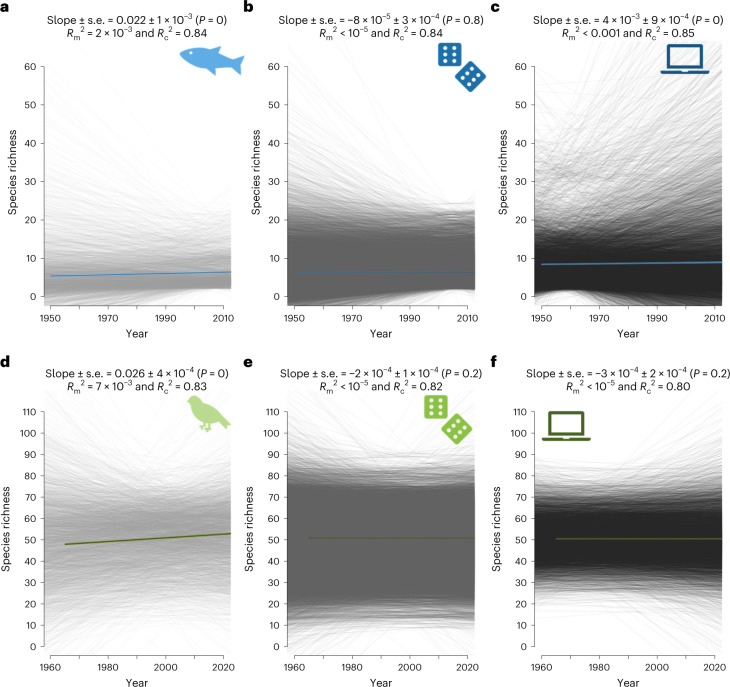
Species richness over time for freshwater fish and breeding birds. **a**–**f**, Species richness over time for freshwater fish (**a**–**c**) and breeding birds (**d**–**f**). Background lines are the empirical (**a**,**d**), null model based (**b**,**e**) and simulated (**c**,**f**) trends in species richness estimated with a linear regression for each individual site. Coloured lines are the output of the LME models (estimate ± s.e.) from which estimates and goodness-of-fit are indicated on each panel. *R*_m_^2^, marginal *R*^2^; *R*_c_^2^, conditional *R*^2^.

Here, we combine observational data and simulations to test whether this imbalance is strong enough to fundamentally shift species richness trends to slopes different from zero by default. We first analysed species richness trends using 3,036 European empirical freshwater fish community time series from the highly curated RivFishTIME dataset^12^ (average duration = 24 years), along with 4,317 time series from the Breeding Bird Survey in North America^13^ (average duration = 37 years; Methods). Across the empirically sampled communities, the average slopes from the linear mixed-effects (LME) model were +0.02 (standard error (s.e.) = 0.001, *P* < 0.001, marginal *R*^2^ = 0.002, conditional *R*^2^ = 0.85) and +0.03 (s.e. = 0.0001, *P* < 0.001, marginal *R*^2^ = 0.007, conditional *R*^2^ = 0.83) for freshwater fish and breeding bird communities, respectively (Fig. 2a,d). The empirical data thus correspond to previous meta-analyses^4,10,14,15^, showing no overall decline in local richness, but rather a small yet significant average increase over time.

**Fig. 3:**
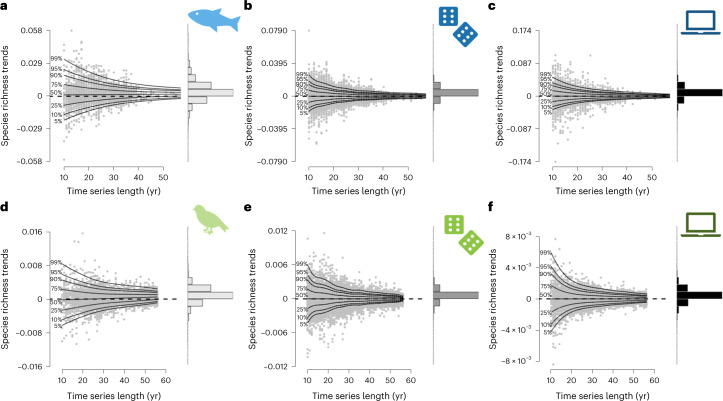
Effect of time series length on species richness trends for observed, randomized and simulated data for riverine fish and breeding birds. **a**–**f**, Effect of time series length on species richness trends for observed (**a**,**d**), randomized (**b**,**e**) and simulated (**c**,**f**) data for riverine fish (**a**–**c**) and breeding birds (**d**–**f**). For each panel, on the right side, the distribution of the species richness trends is represented, and the solid black lines represent percentile curves estimated with GAMLSS.

Shorter time series revealed more variable estimates for slopes and larger standard errors (Fig. 3, Supplementary Figs. 3 and4, and Supplementary Tables 4 and 5). To test whether the positive overall richness trend was driven by short time series only, we used a generalized additive model for location scale and shape (GAMLSS^16^; Methods). While only the variance in species richness trends was affected by time series length for freshwater fish (estimate_slope_ ± s.e. = 1 × 10^−5^ ± 1 × 10^−^^5^, *P* = 0.3; estimate_variance_ ± s.e. = −0.04 ± 2 × 10^−^^3^, *P* < 0.001; *R*^2^ = 0.20), both the mean and the variance in species richness trends were impacted for birds (estimate_slope_ ± s.e. = 1 × 10^−^^5^ ± 1 × 10^−^^6^, *P* < 0.001; estimate_variance_ ± s.e. = −0.03 ± 8 × 10^−^^4^, *P* < 0.001; *R*^2^ = 0.29; Fig. 3a,d). Thus, when dispersal is not strongly constraining communities (for example, avian communities), short time series exhibit a duration-related underestimation bias in the observed trends. While we fully acknowledge the time and money already needed to collect such data^17^, we need to accept that most currently used worldwide long-term datasets actually capture relatively short time series^18,19^. Therefore, our results strongly suggest that short time series potentially underestimate diversity loss, as previously claimed^20^.

We compared these observations with a null model for which we fully randomized the observed yearly chronosequences of species, thereby fully removing temporal autocorrelation from year to year in species dynamics. Such null models are often used to provide a benchmark for a given diversity metric in the absence of driving processes^21^. For both taxa-specific null models, species richness was steady over time (LME, fish: estimate ± s.e. = −8 × 10^−^^5^ ± 3 × 10^−^^4^, *P* = 0.8, marginal *R*^2^ < 0.001, conditional *R*^2^ = 0.84; birds: estimate ± s.e. = −2 × 10^−^^4^ ± 1 × 10^-4^, *P* = 0.2, marginal *R*^2^ < 0.001, conditional *R*^2^ = 0.82; Fig. 2b,e), while the variance was reduced under long time series (fish: estimate_variance_ ± s.e. = −5 × 10^−^^2^ ± 5 × 10^−^^4^, *P* < 0.001, *R*^2^ = 0.30; birds: estimate_variance_ ± s.e. = −4 × 10^−^^2^ ± 3 10^−^^4^, *P* < 0.001, *R*^2^ = 0.48; Fig. 3b,e). However, this classic null model approach is highly unrealistic for biodiversity time series as it allows any species to flip between absence, rare and abundant occurrences, which does not occur in actual populations. In the absence of catastrophic extinctions, the population size at any time point is correlated with the abundance at the previous time step via the specific birth and death rates, resulting in strong temporal autocorrelation under regular monitoring when sampling intervals are not very large compared with generation time.

To analyse whether incorporating temporal autocorrelation matters for null expectations, we simulated 9,999 time series of neutral communities. These simulations matched the empirical observations with respect to mean and variance of time series length and species richness. We derived these time series from a neutral model^22,23^ based on the theory of island biogeography^2^, simulating species occurrences while controlling for equilibrium richness and temporal autocorrelation. We explored a large range of autocorrelations (Supplementary Table 1), but highlight a case with an autocorrelation level matching the observed temporal autocorrelation. Despite being a neutral model, simulated time series for river fish exhibited increased species richness over time (estimate ± s.e. = 4 × 10^−^^3^ ± 9 × 10^−^^4^, *P* < 0.001, marginal *R*^2^ < 0.001, conditional *R*^2^ = 0.85), which suggests that these fish communities are not at equilibrium with their historical context (Fig. 2c,f, Supplementary Figs. 1 and 2, and Supplementary Tables 2 and 3). By contrast, simulated time series for breeding birds did not show a significant deviance from neutral trends (estimate ± s.e. = −3 × 10^−^^4^ ± 2 × 10^−^^4^, *P* = 0.2, marginal *R*^2^ < 0.001, conditional *R*^2^ = 0.80), which may reflect that bird communities are less constrained in their dispersal, allowing stronger rescue effect^24^. The simulated slope of richness over time was significantly independent from time series length (fish: estimate_slope_ ± s.e. = −6 × 10^−^^6^ ± 1 × 10^−^^5^, *P* = 0.6, *R*^2^ = 0.20; birds: estimate_slope_ ± s.e. = 2 × 10^−^^7^ ± 4 × 10^−^^7^, *P* = 0.7, *R*^2^ = 0.48), only variance in species richness trends decreased with longer time series (fish: estimate_variance_ ± s.e. = −5 × 10^−^^2^ ± 9 × 10^−^^4^, *P* < 0.001; birds: estimate_variance_ ± s.e. = −4 × 10^−^^2^ ± 5 × 10^−^^4^, *P* < 0.001; Fig. 3c,f). This pattern holds for most of the settings of autocorrelation and balance between colonization and extinction we have tested (Supplementary Figs. 3 and 4, and Supplementary Tables 4 and 5). Thus, the observed departure from a zero slope for simulated data, especially in the case of riverine fish, is not linked to the empirical time series being too short^25^.

### Net imbalance between colonizations and extinctions

Richness increases are inevitable when population dynamics exhibit strong autocorrelation (for example, strong dispersal limitation), which may mask the true richness trends (expected to be null in the simulations), even for time periods substantially longer than our observations. Our simulations turn the interpretation of the RivFishTIME data around: on average, richness increases, but less than expected from a neutral community with similar autocorrelation. The observed positive trend thus is a negative deviation from the neutral expectation, meaning that colonizations happen slower and/or extinctions faster than needed to balance winners and losers. To test whether the bias towards positive richness trends is based on the imbalance between colonization and extinctions, we compared the cumulative number of colonizations (*C*_cum_) and extinctions (*E*_cum_) over time in observed, randomized and simulated data. We used optimal linear estimation (OLE) models^26,27^ to estimate true colonization and extinction times of each species, as the raw first and last sightings are biased by the finite time frame of the time series. When OLE models estimated that colonizations probably occurred before the observation period and extinctions thereafter, the species was considered persistent. Based on all species, we calculated the net imbalance between colonizations and extinctions (NICE) over time. A perfect balance results in NICE = 0, while positive values indicate colonizations exceeding extinctions and negative values the opposite (Methods).

**Fig. 4:**
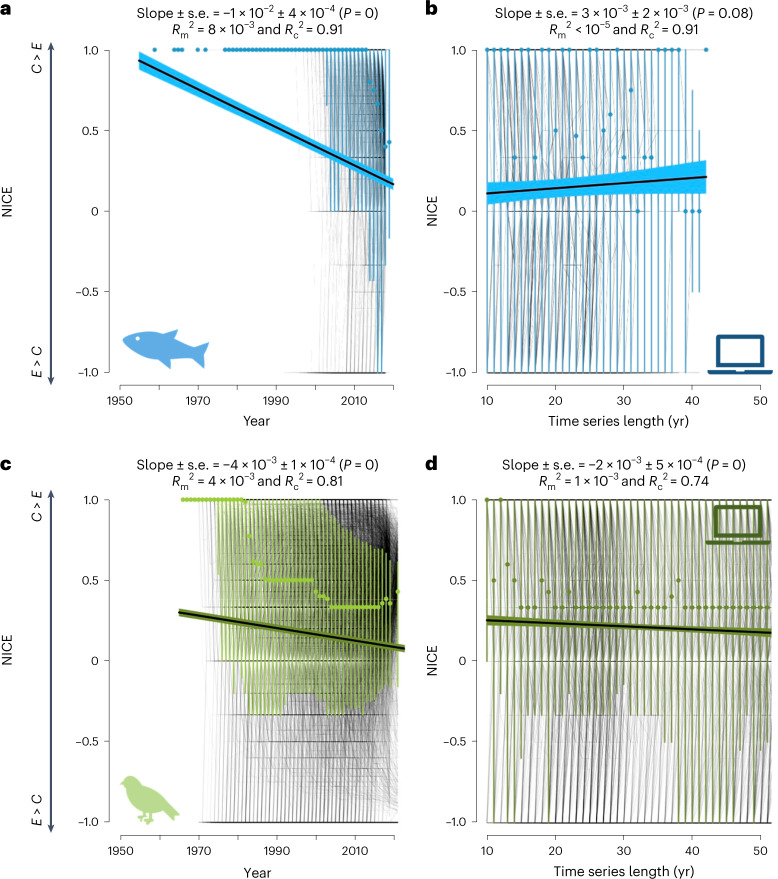
Temporal trends in the imbalance between colonizations and extinctions measured as the NICE metric over time for observed and simulated freshwater fish and breeding birds. **a**–**d**, Temporal trends in the imbalance between colonizations (*C*) and extinctions (*E*) measured as the NICE metric over time for observed (**a**,**c**) and simulated (**b**,**d**) freshwater fish (**a**,**b**) and breeding birds (**c**,**d**) time series. Background lines are the time series while the dark lines are the output of the LME models (estimate ± s.e.) from which estimates and goodness-of-fit are indicated on each panel. Points indicate the median value for each year while the associated bars represent the 25th and 75th quantiles to better represent the distributions of NICE values over time.

Across all time series, final NICE values were positive (fish: mean NICE_observed_ ± s.d. = 0.17 ± 0.8; birds: mean NICE_observed_ ± s.d. = 0.11 ± 0.7) and significantly different from zero (Student’s *t*_fish_ = 83, Student’s t_birds_ = 103, all *P* < 0.001) for both taxonomic groups (Fig. 4). The imbalance slightly decreased over time (LME overall slope of NICE_observed_ over time for fish = −1 × 10^−2^, *P* < 0.001; and birds = −4 × 10^−^^3^, *P* < 0.001; Fig. 4). For simulated data, NICE values decreased over time at a slower rate than observed for birds (estimate_simulated_ = −2 × 10^−^^3^, *P* = 0.08) while even being steady over time for fish (estimate_simulated_ = −3 × 10^−^^3^, *P* < 0.001; Supplementary Figs. 5 and 6). Decreases in NICE values suggest that imbalances between *C*_cum_ and *E*_cum_ might disappear if environmental changes stop. However, the difference between observed and simulated trends in NICE suggests that extinctions are catching up with colonizations faster than predicted, which would ultimately further increase the negative deviation from the neutral prediction.

Our analyses have major implications for our understanding of biodiversity changes, but also for monitoring strategies, assessments and the formulation of conservation targets, including a reinterpretation of the ‘neutral trend in richness’ meta-analyses^4,10,14,15^. If most of the temporal data in these analyses have some degree of autocorrelation coupled with strong dispersal limitation, the reported zero slope does not necessarily imply constant levels of richness, but a deviation trajectory. For fish, this suggests that either colonization does not happen as fast as expected under the extinction regime, or extinction is faster than expected at the level of colonization observed. This turns the main outcome of these meta-analyses into a message of potential biodiversity decline, as the neutral prediction for changes is not necessarily a zero slope, at least for time series that are characterized by ongoing environmental change, such as climate change that changes composition by allowing colonization by ‘winners’ and extinction of ‘losers’.

We used freshwater fish as an empirical example, as they are among the most threatened taxa^28^ and are especially sensitive to their environment, but also strongly constrained by the hydrological network, making escaping unsuitable conditions difficult^29^. We found simulations suggest that this increase in species richness is not fast enough to reflect long-term balanced extinction–colonization dynamics. As fish communities seem to experience sub-optimal, albeit suitable, conditions, exclusion of species is likely to take time, especially if the environment changes marginally, resulting in conditions not too far from the species optimum. The colonizers’ origin was beyond the scope of this paper, but non-native species pose a critical threat to freshwater native communities^30,31^ that can eventually result in increased rates of extinction^32^. Thus, considering species’ origins will probably provide further insights regarding diversity dynamics and the underlying drivers^33^. On the other hand, based on our simulation for avian communities, neutral species richness trends were equal to zero, meaning that North American bird communities are experiencing an actual increase in species number. Birds being good long-distance dispersers, avian community dynamics can be strongly impacted by rescue effects. Thus, extinctions are probably evened out, although new colonizations, for instance, by non-native species, are unlikely to fully compensate for functional loss from the native extinctions^34^. However, also based on neutral predictions, we found that extinctions are catching up with colonizations faster than expected. Thus, although for now bird communities are experiencing an increase in species richness, these temporal dynamics might be hindered by an increasing relative rate in extinctions, ultimately resulting in this increase in species number being only a transient state.

As our simulations show that richness increases by colonization–extinction imbalance are transient, they do not contradict key dynamic equilibrium theories such as the island biogeography theory^2^ and the unified neutral theory of biodiversity and biogeography^1^. However, the more autocorrelated the population dynamics were, the more the imbalance between colonizations and extinctions was critical. We are not the first to report on such extended presence of non-equilibrium richness^35^, but we place this idea into the context of biodiversity response to continuing and unidirectional environmental change (for example, urbanization, climate change). The transient imbalance is likely to be shifted towards colonization and lead to richness gain. This incomplete species sorting over time will be more extensive for more long-lived organisms^36^ and more dispersal constrained taxa, which are thus likely to experience the mismatch between their ecological niche and the environment for longer. However, extinctions will probably eventually catch up with colonizations when environmental conditions stop changing or when further colonization is impaired by the limited size of the species pool^37^.

Delays in trends in species richness can emerge from biases and/or actual biological processes (for example, phenotypic plasticity, use of microrefugia), resulting in imbalance between colonizations and extinctions. Although empirical data can be anywhere along the spectrum—from ecological mechanisms being the only source of bias (for example, extinction debts) to purely methodological biases—the use of neutral baselines to infer temporal trends allows potential sources to be ruled out by having ecologically null predicted trends^19^. In particular, here our neutral model allowed us to compare empirical data with null predictions to draw the following conclusions: (1) fish communities are experiencing a slower increase in diversity than expected; and (2) avian communities are exhibiting an actual increase in species richness with no apparent delays. Complementarily, NICE temporal dynamics can offer us insights regarding the ecological mechanisms underlying delays in trends, namely the imbalance between colonizations and extinctions. For instance, we showed here that although birds are not experiencing delays in species richness changes, this might be a transient pattern, given the negative trends in NICE values over time. The simultaneous use of neutral models and simple yet straightforward metrics such as NICE can allow us to disentangle mechanisms impacting species richness trend estimation.

Providing methods to quantify an accurate baseline to correct species richness trends for their inherent positive bias remains a challenge. Classically, null models remove all temporal autocorrelation in species temporal fluctuations in occurrences^21^. They are used to characterize the impact of long-term environmental changes (for example, climate change) or regular disturbance regimes (for example, tide-related disturbances, El Niño cycles) on communities and their diversity^38–40^. Although these null models provide a baseline in which environmental forcing, dispersal and species interaction effects are all simultaneously removed^21^, in the context of compositional time series they delete a key constraint to our understanding of biodiversity trends: the temporal dependence of species abundances. Therefore, our simulations are neutral as species do not interact, but their dynamics are constrained by changes in population growth rates. While the null model with no temporal autocorrelation shows expected species richness trends equal to zero, the temporal constraint on population dynamics leads to a new baseline of increasing species richness, even when there is no environmental forcing. Additionally, the environmental trends are often neither white noise nor random walks, but show some aspect of autocorrelation as well^41^. The bias introduced to richness trends by the difference between colonization and extinction timing cannot be remedied with a single correction factor, as the amount of bias will differ between sites and organisms. More isolated sites will show less bias towards immigration^6^, while longer-lived organisms will show more extensive extinction debt as individual generations persist longer^36^. We propose here the analysis of the NICE metric as a tool to—at least—estimate the extent of this bias, which allows comparing the contribution of trends pre-imposed by continuous environmental changes with the overall trends across empirical time series.

## Methods

### Empirical time series

To describe community dynamics over time, we used two highly curated databases. First, the RivFishTIME database, which gathers freshwater fish abundance time series^12^. We focused our analysis on 3,036 European time series with at least 10 years sampled. The final dataset comprised time series starting in 1951 and finishing in 2019 with 12 sampled years on average (s.d. = 6.6 years). Second, we used the North American Breeding Bird Survey database^13^ which represents 4,317 time series sampled at least 10 times, comprising time series starting in 1966 and finishing in 2021 (29 sampled years on average ± 12.5 years).

### NICE over time

As initial metrics, we estimated colonization and extinction events for each species in each time series using OLE models^26,27^, using the OLE function from the sExtinct package^42^, allowing for a more conservative quantification of colonization and extinction times. Although OLE models do not account for abundance dynamics, the key advantage of using them is not to rely only on the first and last sighting of a species, but rather to infer how much longer the species is likely to have persisted before and after the known occurrences. Any events (that is, colonizations and extinctions) happening outside the sampled time window of the focal community were disregarded. Thus, extinctions can theoretically happen more often than colonizations if the latter happen earlier than the beginning of the sampling time.

To compare the colonization versus extinction dynamics, we computed the NICE for each sampled year. The NICE metric quantifies the cumulative magnitude and direction of potential imbalance between local colonizations and extinctions in a comparable way across time series, and is calculated as follows:

$${{\mathrm{NICE}}}=\,\frac{{C}_{{{\mathrm{cum}}}}-{E}_{{{\mathrm{cum}}}}}{{C}_{{{\mathrm{cum}}}}+{E}_{{{\mathrm{cum}}}}}$$

Positive values indicate faster colonizations than extinctions (that is, delayed net loss), while negative values suggest slower colonizations than extinctions (that is, delayed net gain). Moreover, we estimated trends in log-transformed species richness using linear models and investigated the relationship between these trends and time series length.

### Simulated data

We used a model based on the theory of island biogeography to generate artificial data akin to the studied datasets. This model tracks the change in species richness in a site over time as follows:

$$\frac{{\mathrm{d}}{S}_{{\mathrm{S}}}}{{{\mathrm{d}}t}}\,=\,c({S}_{{\mathrm{P}}}\,-\,{S}_{{\mathrm{S}}})\,-\,e{S}_{{\mathrm{S}}}$$

where *S*_S_ is the number of species in a site at a time point *t*, *S*_P_ the number of species in the pool, and *c* and *e* are colonization and extinction rates, respectively. The R package island^23^ implements the dynamics of this model, of which its equilibrium richness is known to be $\frac{c}{c+e}{S}_{{\mathrm{P}}}$ and its temporal autocorrelation has been shown to be exp[−(*c* + *e*) Δ*t*]^22^, where Δ*t* is the time between two consecutive samplings (which defaults to 1 for simplicity in our case). The above model is easily solved for a single species^43^, leading to a Markov chain with two states for the species, which can be either present (1) or absent (0), and known transition probabilities between these states. Assuming that all species are equivalent and independent, we can obtain the temporal dynamics of a community, given its initial richness, number of species in the pool, and colonization and extinction rates. These rates have been based on the empirical data as the number of colonization events over a time series divided by the length of the time series. Thus, we simulated 9,999 time series of presence–absence data using function PA_simulation from R package island, for a species pool randomly drawn from the distribution of total number of species observed for a given time series, and time series length and initial species richness sampled at random from the observed distribution of these values in the empirical databases. As a null model, we assumed that *c* = *e*, that is, the probability of any species of being present was 0.5, and a varying degree of temporal autocorrelation, which allowed us to examine the effect of transient dynamics on the model. The simulated data presented in the main text refers to an autocorrelation based on observed *c* and *e* in the empirical data. Moreover, we explored different imbalances between colonizations and extinctions. We focused only on the balanced rates in the main text, but results based on non-equal rates can be found in the Supplementary Information.

### Effect of time series length on species richness trends over time

To assess the potential effect of time series length on log-transformed species richness trends, we used a GAMLSS^16^, which offers a highly flexible framework with regard to the response variable distribution while allowing for fitting distribution parameters as a function of the independent variable. Thus, both the mean and the variance of first the species richness trends and second the NICE values can be modelled as a linear function of time.

### Reporting summary

Further information on research design is available in the Nature Portfolio Reporting Summary linked to this article.

## Supplementary information

Supplementary Information Supplementary Figs. 1–6 and Tables 1–5.

Reporting Summary

Peer Review File

## Data Availability

All data used in this study were attained from publicly available databases and the sources of all data and links to databases are provided at the appropriate section in the manuscript. Processed data are available on GitHub at https://github.com/Lucie-KCZ/NeutralDynamics.

## Code availability

The code is available on GitHub at https://github.com/Lucie-KCZ/NeutralDynamics.
