# Supplementary material for: Biodiversity time series are biased towards increasing species richness in changing environments
Source: Nat Ecol Evol. 2023 Jun 5;7(7):994–1001. doi: 10.1038/s41559-023-02078-w (PMC10333117; doi:10.1038/s41559-023-02078-w)

nature ecology & evolution

Article https://doi.org/10.1038/s41559-023-02078-w

Biodiversity time series are biased towards increasing species richness in changing environments

In the format provided by the authors and unedited

1. DESCRIPTION OF ALL SETTINGS WITH WHICH SIMULATIONS HAVE BEEN RUN 2

a. Different autocorrelation settings..2

b. Different imbalance between colonisation and extinction rates 3

2. SPECIES RICHNESS TRENDS ACROSS SIMULATION SETTINGS 4

a. RivFishTIME settings...4

b. BBS settings...6

3. EFFECT OF TIME SERIES LENGTH ON SPECIES RICHNESS TRENDS ACROSS SIMULATION SETTINGS.. 8

a. RivFishTIME settings...8

b. BBS settings...10

4. NICE ACROSS SIMULATION SETTINGS 12

a. RivFishTIME settings...12

b. BBS settings...13

1. Description of all settings with which simulations have been run

    a. *Different autocorrelation settings*

Different temporal autocorrelation settings have been tested through different simulations. Autocorrelation is based on the colonization and extinction rates: the higher the rates are, the weaker is the temporal autocorrelation (i.e., the communities are more dynamics and less predictable). In particular, temporal autocorrelation is measured on the Markov chain (the temporal series of simulated absences and presences) for a single species, and according to Puljapurkar (1997) lag_{-1} temporal autocorrelation is equal to $1 – (T_{01} - T_{10})$, with T_{01} and T_{10} being the transition probabilities of the Markov chain corresponding to extinction and colonization, respectively. We would highlight here that temporal autocorrelation is measured on the series of presences and absences, that in turn determine the number of colonization and extinction events on that time series. So, temporal autocorrelation in these simulations refer to the maintenance of absences and presences in the temporal series, that is, the probability of not observing a change of state (1 - present, 0 - absent) in the series. It is clear that when $c = e$, and therefore $T_{01} = T_{10}$, the expected time that both absences and presences are maintained in the system is equal. However, when $c \neq e$, the expected time of presence and absence are necessarily different, but autocorrelation is still measured as a property of the whole series.

First, we ran simulations using colonisation rates estimated from the empirical data (fish: 0.41 $species.year^{-1}$; birds: 0.99 $species.year^{-1}$). Using these rates, the autocorrelation is as observed in the empirical data. By multiplying the observed rates by a constant, we could simulate stronger (i.e., constant > 1) and weaker (i.e., constant < 1) temporal autocorrelation than the observed one. It is thus important to keep in mind that variation in autocorrelation is relative here. For all the following figures, the constant is 0.001 for the panels A, F, K, P and U (i.e., very low relative autocorrelation); 0.1 for the panels B, G, L, Q and V (i.e., low autocorrelation); 1 for the panels C, H, M, R and W (i.e., observed temporal autocorrelation); 10 for the panels D, I, N, S and X (i.e., high temporal autocorrelation) and, 25 for the panels E, J, O, T and Y (i.e., very high autocorrelation). Although our results were about the relative temporal autocorrelation, they allowed us to gain insights about the absolute temporal autocorrelation as well as increased relative autocorrelation still result in increase in absolute temporal autocorrelation of the first order (i.e., correlation between x_t and x_{t-1}).

Table S1: Range of the Spearman's correlations between SR at time t and time t-1 (i.e., temporal autocorrelation of the first order) across the different relative autocorrelation settings.

Relative autocorrelation	Fish		Birds	
	Minimum	Maximum	Minimum	Maximum
Very low	-0.14	-0.14	-0.06	-0.05
Low	-0.15	-0.14	-0.06	-0.05
As observed	0.06	0.23	0.02	0.14
High	0.52	0.66	0.63	0.87
Very high	0.58	0.69	0.76	0.92

Reference: Tuljapurkar S. (1997). Stochastic Matrix Models. In: *Structured Population Models in Marine, Terrestrial, and Freshwater Systems, pages 59-87. Springer US, Boston, MA.*

b. Different imbalance between colonisation and extinction rates

We have run different sets of simulations testing for different (im)balances between colonization and extinction rates. The five settings are the following:

- More colonizations: COL = EXT * ¼ (coded as +2 for the correlation tests)
- Slightly more colonizations: COL = EXT * ¾ (coded as +1 for the correlation tests)
- Balance: COL = EXT (coded as 0 for the correlation tests)
- Slightly more extinctions: EXT = COL * ¾ (coded as -1 for the correlation tests)
- More extinctions: EXT = COL * ¼ (coded as -2 for the correlation tests)

2. Species richness trends across simulation settings

a. RivFishTIME settings

Species richness trends were, albeit significantly, only slightly correlated with the relative autocorrelation ($\rho_{spearman}$ = 0.051, p < 0.001, n = 1197925) but positively, although weakly, with the imbalance between colonization and extinction rates ($\rho_{spearman}$ = 0.17, p < 0.001, n = 1197925).

Fig S1 – Species richness trends for the different simulation settings. Lines are a single simulation. The first line (A to E) represents a strong imbalance in favor of colonizations, the second (F to J) a slight imbalance in favor of colonizations, the middle one (K to O) a perfect balance between colonization and extinction rates, the fourth (P to T) a slight imbalance in favor of extinction and the last one (U to Y) a strong imbalance in favor of extinction. The first column (A, F, K, P and U) represents a very low temporal autocorrelation, the second (B, G, L, Q and V) a low temporal autocorrelation, the middle one (C, H, M, R and W) the settings for observed autocorrelation (i.e., based on observed rates), the fourth (D, I, N, S and X) a high temporal autocorrelation and the last column (E, J, O, T and Y) a very strong temporal autocorrelation.

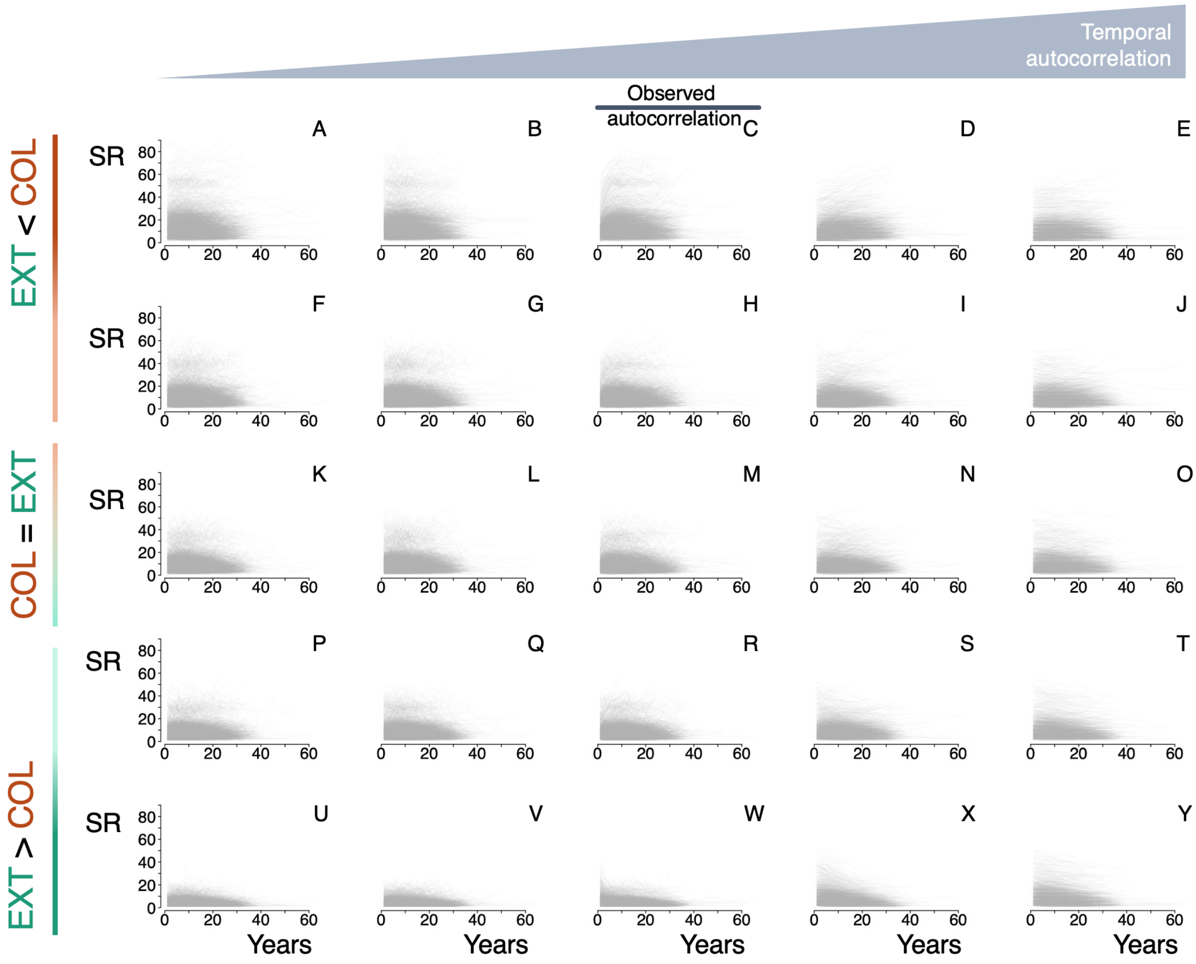

Table S2 – Species richness trends based on linear mixed effect with the simulation id as the random effect.

Autocorrelation	Balance	Estimate	SE	*pvalue*	R^2	
					marginal	conditional
	COL >> EXT	-2 10^{-4}	7 10^{-4}	0.8	8 10^{-8}	0.95
	COL > EXT	-8 10^{-4}	1 10^{-3}	0.5	1 10^{-6}	0.88
Very low	COL = EXT	-4 10^{-4}	1 10^{-3}	0.7	6 10^{-7}	0.84
	COL < EXT	1 10^{-3}	1 10^{-2}	0.5	3 10^{-6}	0.81
	COL << EXT	-2 10^{-3}	2 10^{-3}	0.4	9 10^{-6}	0.62
	COL >> EXT	9 10^{-4}	7 10^{-4}	0.2	2 10^{-6}	0.95
	COL > EXT	-1 10^{-3}	1 10^{-3}	0.3	4 10^{-6}	0.88
Low	COL = EXT	-6 10^{-5}	1 10^{-3}	1	1 10^{-8}	0.85
	COL < EXT	5 10^{-4}	1 10^{-3}	0.7	7 10^{-7}	0.81
	COL << EXT	3 10^{-3}	2 10^{-3}	0.1	3 10^{-8}	0.62
	COL >> EXT	**4 10^{-2}**	**8 10^{-4}**	**0**	**4 10^{-3}**	**0.94**
	COL > EXT	**1 10^{-2}**	**1 10^{-3}**	**0**	**5 10^{-4}**	**0.88**
As observed	COL = EXT	**6 10^{-3}**	**1 10^{-3}**	**0**	**9 10^{-5}**	**0.85**
	COL < EXT	-2 10^{-3}	1 10^{-3}	0.09	1 10^{-5}	0.83
	COL << EXT	**-6 10^{-2}**	**2 10^{-3}**	**0**	**1 10^{-2}**	**0.66**
	COL >> EXT	**1 10^{-1}**	**8 10^{-4}**	**0**	**4 10^{-2}**	**0.93**
	COL > EXT	**5 10^{-2}**	**9 10^{-4}**	**0**	**9 10^{-3}**	**0.90**
High	COL = EXT	**2 10^{-2}**	**1 10^{-3}**	**0**	**2 10^{-3}**	**0.89**
	COL < EXT	**3 10^{-3}**	**1 10^{-3}**	**0**	**4 10^{-5}**	**0.89**
	COL << EXT	**-9 10^{-2}**	**9 10^{-4}**	**0**	**2 10^{-2}**	**0.91**
	COL >> EXT	**8 10^{-2}**	**6 10^{-4}**	**0**	**2 10^{-2}**	**0.96**
	COL > EXT	**4 10^{-2}**	**7 10^{-4}**	**0**	**7 10^{-3}**	**0.94**
Very high	COL = EXT	**3 10^{-2}**	**8 10^{-4}**	**0**	**3 10^{-3}**	**0.94**
	COL < EXT	**7 10^{-3}**	**7 10^{-4}**	**0**	**1 10^{-4}**	**0.94**
	COL << EXT	**-5 10^{-2}**	**6 10^{-4}**	**0**	**7 10^{-3}**	**0.96**

b. BBS settings

Species richness trends were, albeit significantly, only slightly correlated with the relative autocorrelation ($\rho_{spearman}$ = -0.063, p < 0.001, n = 2810824) but positively, although weakly, with the imbalance between colonization and extinction rates ($\rho_{spearman}$ = 0.37, p < 0.001, n = 2810824).

Fig S2 – Species richness trends across the different simulation settings. Lines are a single simulation. The first line (A to E) represents a strong imbalance in favor of colonizations, the second (F to J) a slight imbalance in favor of colonizations, the middle one (K to O) a perfect balance between colonization and extinction rates, the fourth (P to T) a slight imbalance in favor of extinction and the last one (U to Y) a strong imbalance in favor of extinction. The first column (A, F, K, P and U) represents a very low temporal autocorrelation, the second (B, G, L, Q and V) a low temporal autocorrelation, the middle one (C, H, M, R and W) the settings for observed autcorrelation (i.e., based on observed rates), the fourth (D, I, N, S and X) a high temporal autocorrelation and the last column (E, J, O, T and Y) a very strong temporal autocorrelation.

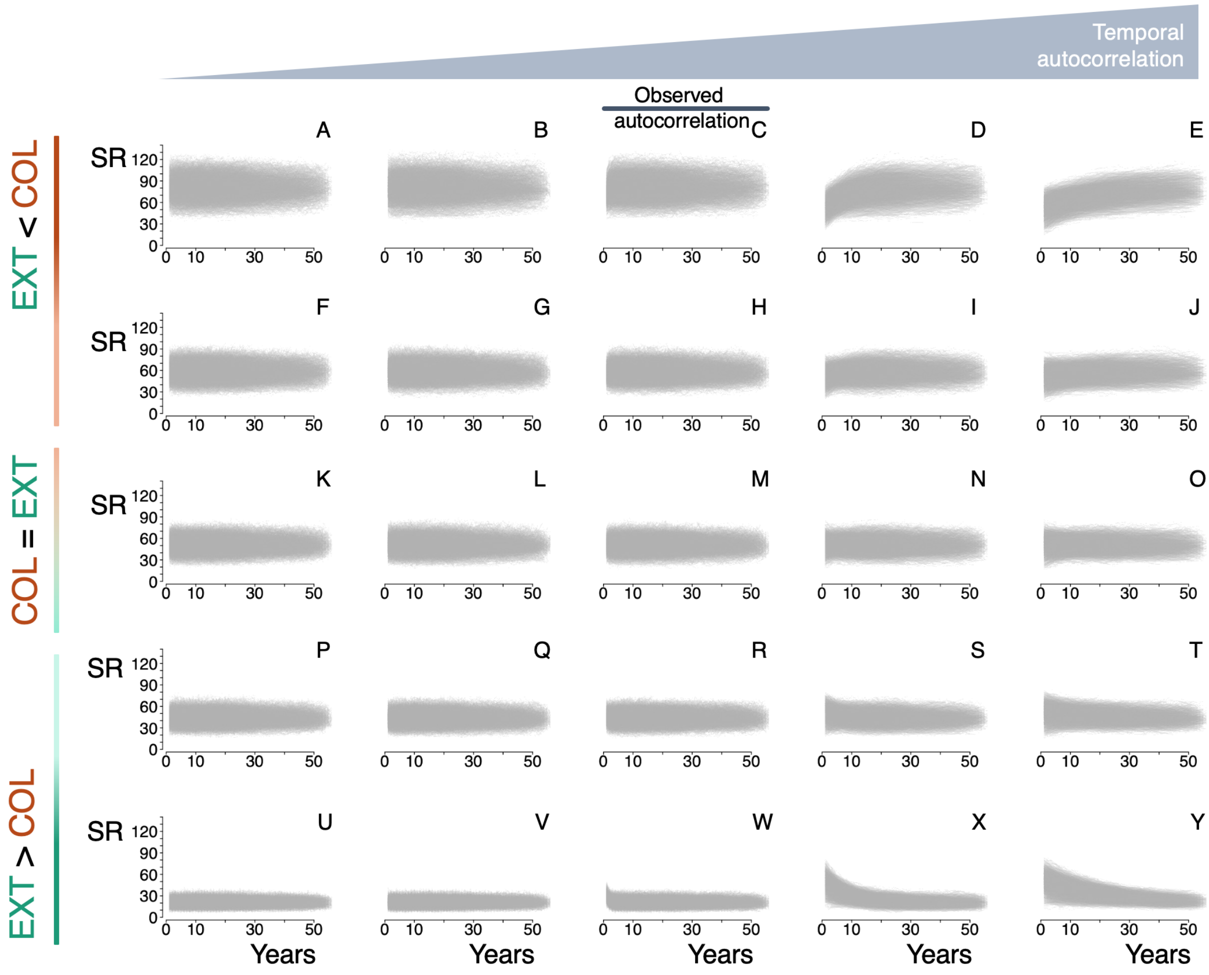

Table S3 – Species richness trends based on linear mixed effect models with the simulation id as a random effect.

Autocorrelation	Balance	Estimate	SE	*pvalue*	R^2	
					marginal	conditional
Very low	COL >> EXT	$-2\ 10^{-4}$	$2\ 10^{-4}$	0.2	$1\ 10^{-6}$	0.94
	COL > EXT	$-3\ 10^{-4}$	$3\ 10^{-4}$	0.3	$2\ 10^{-6}$	0.84
	COL = EXT	$3\ 10^{-4}$	$3\ 10^{-4}$	0.4	$1\ 10^{-6}$	0.79
	COL < EXT	$-5\ 10^{-4}$	$4\ 10^{-4}$	0.2	$4\ 10^{-6}$	0.74
	COL << EXT	$-2\ 10^{-4}$	$6\ 10^{-4}$	0.8	$4\ 10^{-7}$	0.48
Low	COL >> EXT	$2\ 10^{-5}$	$2\ 10^{-4}$	0.9	$8\ 10^{-9}$	0.94
	COL > EXT	$-1\ 10^{-4}$	$3\ 10^{-4}$	0.7	$3\ 10^{-7}$	0.84
	COL = EXT	$-5\ 10^{-3}$	$4\ 10^{-4}$	0.9	$5\ 10^{-8}$	0.80
	COL < EXT	$-3\ 10^{-3}$	$4\ 10^{-4}$	0.5	$2\ 10^{-6}$	0.74
	COL << EXT	$-2\ 10^{-4}$	$6\ 10^{-4}$	0.8	$3\ 10^{-7}$	0.49
As observed	COL >> EXT	**$6\ 10^{-3}$**	**$2\ 10^{-4}$**	**0**	**$9\ 10^{-4}$**	**0.93**
	COL > EXT	**$8\ 10^{-4}$**	**$3\ 10^{-4}$**	**0.009**	**$1\ 10^{-5}$**	**0.83**
	COL = EXT	$6\ 10^{-5}$	$3\ 10^{-4}$	0.9	$7\ 10^{-8}$	0.80
	COL < EXT	**$-2\ 10^{-3}$**	**$4\ 10^{-4}$**	**0**	**$1\ 10^{-4}$**	**0.73**
	COL << EXT	**$-2\ 10^{-2}$**	**$7\ 10^{-4}$**	**0**	**$7\ 10^{-3}$**	**0.47**
High	COL >> EXT	**$8\ 10^{-2}$**	**$3\ 10^{-4}$**	**0**	**0.12**	**0.79**
	COL > EXT	**$2\ 10^{-2}$**	**$3\ 10^{-4}$**	**0**	**$7\ 10^{-3}$**	**0.77**
	COL = EXT	**$-5\ 10^{-3}$**	**$4\ 10^{-4}$**	**0**	**$6\ 10^{-4}$**	**0.74**
	COL < EXT	**$-3\ 10^{-2}$**	**$4\ 10^{-4}$**	**0**	**$2\ 10^{-3}$**	**0.69**
	COL << EXT	**$-2\ 10^{-1}$**	**$7\ 10^{-4}$**	**0**	**0.37**	**0.66**
Very high	COL >> EXT	**$1\ 10^{-1}$**	**$3\ 10^{-4}$**	**0**	**0.21**	**0.81**
	COL > EXT	**$3\ 10^{-2}$**	**$3\ 10^{-4}$**	**0**	**$2\ 10^{-2}$**	**0.76**
	COL = EXT	**$-5\ 10^{-3}$**	**$4\ 10^{-4}$**	**0**	**$7\ 10^{-4}$**	**0.73**
	COL < EXT	**$-4\ 10^{-2}$**	**$4\ 10^{-4}$**	**0**	**$4\ 10^{-2}$**	**0.73**
	COL << EXT	**$-2\ 10^{-1}$**	**$4\ 10^{-4}$**	**0**	**0.41**	**0.89**

3. Effect of time series length on species richness trends across simulation settings

a. RivFishTIME settings

Fig S3 – Species richness trends in response to time series length across simulation settings. The different quantiles (i.e., 99%, 95%, 90%, 75%, 50%, 25%, 10% and 5%) are represented by the black lines and dots represent a signle simulation. The first line (A to E) represents a strong imbalance in favor of colonizations, the second (F to J) a slight imbalance in favor of colonizations, the middle one (K to O) a perfect balance between colonization and extinction rates, the fourth (P to T) a slight imbalance in favor of extinction and the last one (U to Y) a strong imbalance in favor of extinction. The first column (A, F, K, P and U) represents a very low temporal autocorrelation, the second (B, G, L, Q and V) a low temporal autocorrelation, the middle one (C, H, M, R and W) the settings for observed autcorrelation (i.e., based on observed rates), the fourth (D, I, N, S and X) a high temporal autocorrelation and the last column (E, J, O, T and Y) a very strong temporal autocorrelation.

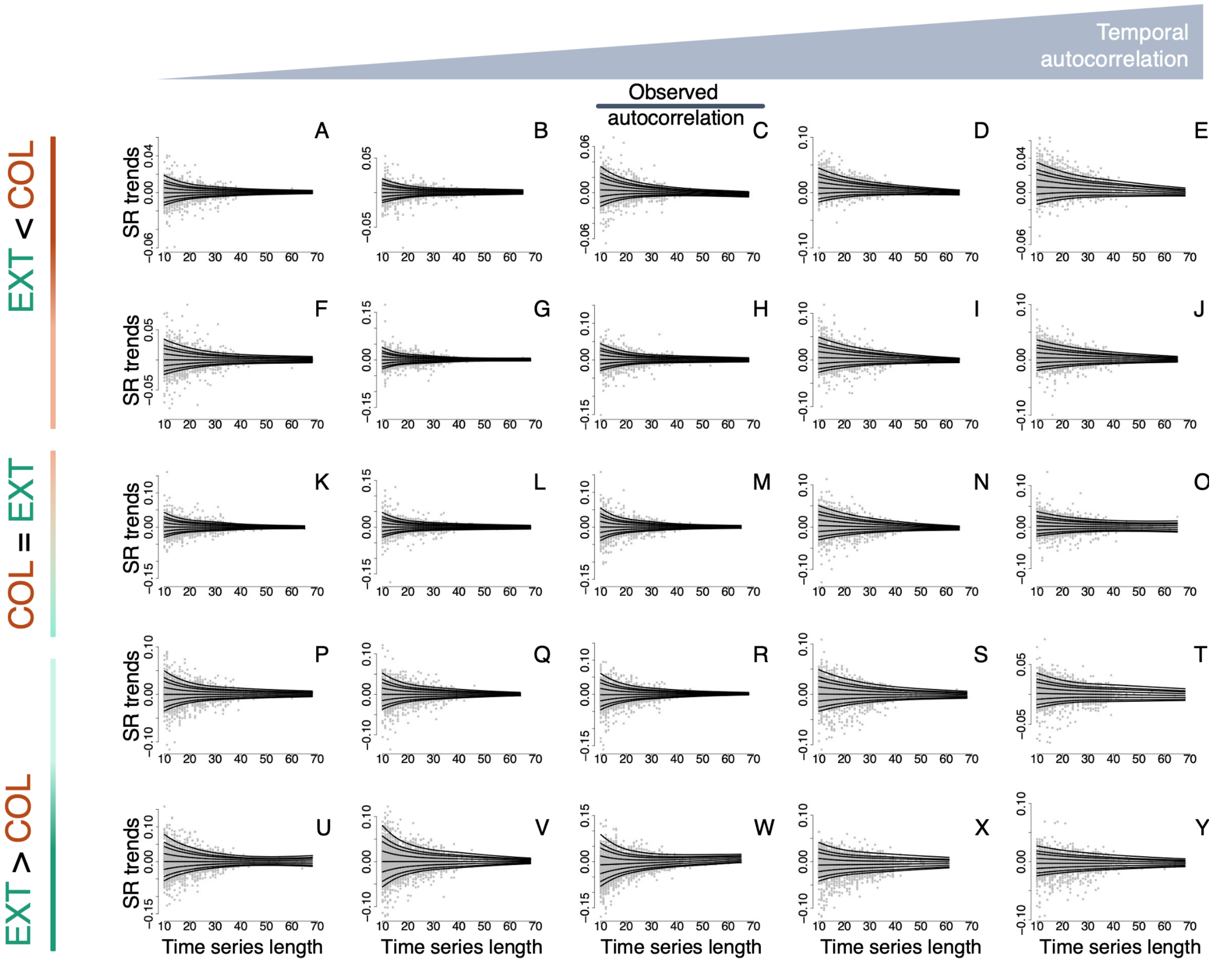

Table S4 – Results from the GAMLSS models on the effect of time series length on species richness trends. The effect of time series length was tested for both the mean trends and the variance.

Autocor.	Balance	Mean			Variance			
		Estimate	SE	*pvalue*	Estimate	SE	*pvalue*	R^2
Very low	COL >> EXT	9 10^{-6}	5 10^{-6}	0.06	-5 10^{-2}	1 10^{-3}	0	0.26
	COL > EXT	-8 10^{-6}	1 10^{-5}	0.5	-5 10^{-2}	1 10^{-3}	0	0.22
	COL = EXT	-2 10^{-5}	1 10^{-5}	0.05	-4 10^{-2}	1 10^{-3}	0	0.24
	COL < EXT	7 10^{-7}	2 10^{-5}	1	**-5 10^{-2}**	**1 10^{-3}**	**0**	0.26
	COL << EXT	-2 10^{-5}	2 10^{-5}	0.4	**-5 10^{-2}**	**1 10^{-3}**	**0**	0.26
Low	COL >> EXT	8 10^{-6}	7 10^{-6}	0.3	**-5 10^{-2}**	**1 10^{-3}**	**0**	0.26
	COL > EXT	9 10^{-6}	1 10^{-5}	0.4	**-5 10^{-2}**	**1 10^{-3}**	**0**	0.29
	COL = EXT	**-3 10^{-5}**	**2 10^{-5}**	**0.04**	**-5 10^{-2}**	**1 10^{-3}**	**0**	0.28
	COL < EXT	2 10^{-6}	1 10^{-5}	0.9	**-5 10^{-2}**	**1 10^{-3}**	**0**	0.25
	COL << EXT	6 10^{-6}	2 10^{-5}	0.8	**-5 10^{-2}**	**1 10^{-3}**	**0**	0.26
As observed	COL >> EXT	**-1 10^{-4}**	**3 10^{-2}**	**0**	**-5 10^{-2}**	**1 10^{-3}**	**0**	0.32
	COL > EXT	**-4 10^{-5}**	**1 10^{-5}**	**0.007**	**-5 10^{-2}**	**1 10^{-3}**	**0**	0.27
	COL = EXT	-1 10^{-5}	1 10^{-5}	0.4	**-5 10^{-2}**	**1 10^{-3}**	**0**	0.33
	COL < EXT	4 10^{-5}	2 10^{-5}	0.1	**-6 10^{-2}**	**1 10^{-3}**	**0**	0.30
	COL << EXT	**3 10^{-4}**	**3 10^{-5}**	**0**	**-5 10^{-2}**	**1 10^{-3}**	**0**	0.30
High	COL >> EXT	**-2 10^{-4}**	**1 10^{-5}**	**0**	**-4 10^{-2}**	**1 10^{-3}**	**0**	0.21
	COL > EXT	**-1 10^{-4}**	**2 10^{-5}**	**0**	**-4 10^{-2}**	**1 10^{-3}**	**0**	0.17
	COL = EXT	**-1 10^{-4}**	**2 10^{-5}**	**0**	**-4 10^{-2}**	**1 10^{-3}**	**0**	0.17
	COL < EXT	**-4 10^{-5}**	**2 10^{-5}**	**0.02**	**-4 10^{-2}**	**1 10^{-3}**	**0**	0.15
	COL << EXT	**6 10^{-5}**	**2 10^{-5}**	**0.007**	**-3 10^{-2}**	**1 10^{-3}**	**0**	0.10
Very high	COL >> EXT	**-1 10^{-4}**	**1 10^{-5}**	**0**	**-3 10^{-2}**	**1 10^{-3}**	**0**	0.14
	COL > EXT	**-7 10^{-5}**	**1 10^{-5}**	**0**	**-3 10^{-2}**	**1 10^{-3}**	**0**	0.12
	COL = EXT	**-7 10^{-5}**	**1 10^{-5}**	**0**	**-3 10^{-2}**	**1 10^{-3}**	**0**	0.10
	COL < EXT	**-6 10^{-5}**	**2 10^{-5}**	**0**	**-3 10^{-2}**	**1 10^{-3}**	**0**	0.10
	COL << EXT	-1 10^{-6}	1 10^{-5}	0.9	**-2 10^{-2}**	**1 10^{-3}**	**0**	0.07

b. BBS settings

Fig S4 – Species richness trends in response to time series length across simulation settings. The different quantiles (i.e., 99%, 95%, 90%, 75%, 50%, 25%, 10% and 5%) are represented by the black lines and dots represent a signle simulation. The first line (A to E) represents a strong imbalance in favor of colonizations, the second (F to J) a slight imbalance in favor of colonizations, the middle one (K to O) a perfect balance between colonization and extinction rates, the fourth (P to T) a slight imbalance in favor of extinction and the last one (U to Y) a strong imbalance in favor of extinction. The first column (A, F, K, P and U) represents a very low temporal autocorrelation, the second (B, G, L, Q and V) a low temporal autocorrelation, the middle one (C, H, M, R and W) the settings for observed autcorrelation (i.e., based on observed rates), the fourth (D, I, N, S and X) a high temporal autocorrelation and the last column (E, J, O, T and Y) a very strong temporal autocorrelation.

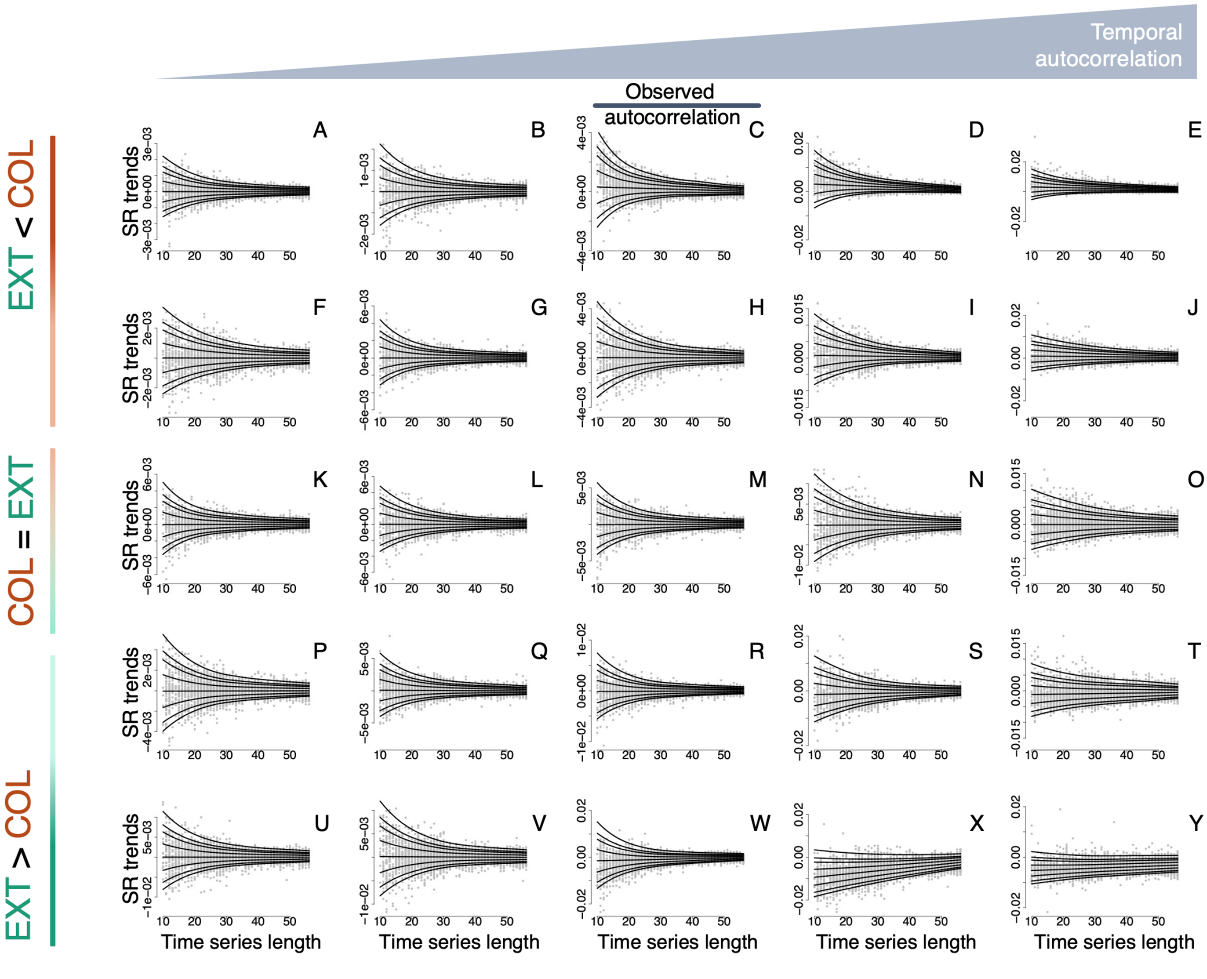

Table S5 – Results from the GAMLSS models on the effect of time series length on species richness trends. The effect of time series length was tested for both the mean trends and the variance.

Autocor.	Balance	Mean			Variance			
		Estimate	SE	*pvalue*	Estimate	SE	*pvalue*	R^2
Very low	COL >> EXT	$-6\ 10^{-8}$	$3\ 10^{-7}$	0.8	**$-4\ 10^{-2}$**	**$7\ 10^{-4}$**	**0**	0.48
	COL > EXT	$-3\ 10^{-7}$	$5\ 10^{-7}$	0.6	**$-4\ 10^{-2}$**	**$7\ 10^{-4}$**	**0**	0.42
	COL = EXT	$6\ 10^{-7}$	$6\ 10^{-7}$	0.3	**$-4\ 10^{-2}$**	**$7\ 10^{-4}$**	**0**	0.46
	COL < EXT	$7\ 10^{-7}$	$7\ 10^{-7}$	0.3	**$-4\ 10^{-2}$**	**$7\ 10^{-4}$**	**0**	0.44
	COL << EXT	$-4\ 10^{-7}$	$1\ 10^{-6}$	0.8	**$-4\ 10^{-2}$**	**$7\ 10^{-4}$**	**0**	0.44
Low	COL >> EXT	$-1\ 10^{-8}$	$3\ 10^{-7}$	1	**$-4\ 10^{-2}$**	**$7\ 10^{-4}$**	**0**	0.45
	COL > EXT	$-2\ 10^{-7}$	$5\ 10^{-7}$	0.7	**$-4\ 10^{-2}$**	**$7\ 10^{-4}$**	**0**	0.47
	COL = EXT	$-3\ 10^{-7}$	$6\ 10^{-7}$	0.6	**$-4\ 10^{-2}$**	**$7\ 10^{-4}$**	**0**	0.46
	COL < EXT	**$-2\ 10^{-6}$**	**$7\ 10^{-7}$**	**0**	**$-4\ 10^{-2}$**	**$7\ 10^{-4}$**	**0**	0.46
	COL << EXT	**$-3\ 10^{-6}$**	**$1\ 10^{-6}$**	**0.02**	**$-4\ 10^{-2}$**	**$7\ 10^{-4}$**	**0**	0.47
As observed	COL >> EXT	**$-6\ 10^{-6}$**	**$4\ 10^{-7}$**	**0**	**$-4\ 10^{-2}$**	**$7\ 10^{-4}$**	**0**	0.59
	COL > EXT	$-5\ 10^{-7}$	$5\ 10^{-7}$	0.3	**$-4\ 10^{-2}$**	**$7\ 10^{-4}$**	**0**	0.49
	COL = EXT	$9\ 10^{-7}$	$6\ 10^{-7}$	0.1	**$-4\ 10^{-2}$**	**$7\ 10^{-4}$**	**0**	0.50
	COL < EXT	**$3\ 10^{-6}$**	**$8\ 10^{-7}$**	**0**	**$-4\ 10^{-2}$**	**$7\ 10^{-4}$**	**0**	0.54
	COL << EXT	**$3\ 10^{-5}$**	**$2\ 10^{-6}$**	**0**	**$-4\ 10^{-2}$**	**$7\ 10^{-4}$**	**0**	0.58
High	COL >> EXT	**$-5\ 10^{-5}$**	**$2\ 10^{-6}$**	**0**	**$-4\ 10^{-2}$**	**$7\ 10^{-4}$**	**0**	0.62
	COL > EXT	**$-1\ 10^{-5}$**	**$2\ 10^{-6}$**	**0**	**$-4\ 10^{-2}$**	**$7\ 10^{-4}$**	**0**	0.50
	COL = EXT	$3\ 10^{-6}$	$2\ 10^{-6}$	0.07	**$-4\ 10^{-2}$**	**$7\ 10^{-4}$**	**0**	0.47
	COL < EXT	**2 10-5**	**$2\ 10^{-6}$**	**0**	**$-4\ 10^{-2}$**	**$7\ 10^{-4}$**	**0**	0.48
	COL << EXT	**$2\ 10^{-4}$**	**$3\ 10^{-6}$**	**0**	**$-3\ 10^{-2}$**	**$7\ 10^{-4}$**	**0**	0.48
Very high	COL >> EXT	**$-4\ 10^{-5}$**	**$2\ 10^{-6}$**	**0**	**$-3\ 10^{-2}$**	**$7\ 10^{-4}$**	**0**	0.43
	COL > EXT	**$-1\ 10^{-5}$**	**$2\ 10^{-6}$**	**0**	**$-3\ 10^{-2}$**	**$7\ 10^{-4}$**	**0**	0.30
	COL = EXT	$-7\ 10^{-7}$	$2\ 10^{-6}$	0.7	**$-3\ 10^{-2}$**	**$7\ 10^{-4}$**	**0**	0.28
	COL < EXT	**$1\ 10^{-5}$**	**$2\ 10^{-6}$**	**0**	**$-3\ 10^{-2}$**	**$7\ 10^{-4}$**	**0**	0.23
	COL << EXT	**$5\ 10^{-5}$**	**$2\ 10^{-6}$**	**0**	**$-1\ 10^{-2}$**	**$7\ 10^{-4}$**	**0**	0.13

4. NICE across simulation settings

a. RivFishTIME settings

Final NICE values were not correlated with the relative autocorrelation ($\rho_{spearman}$ = -0.016, p = 0.9, n = 124975) while being strongly indicative of the imbalance between colonization and extinction rates ($\rho_{spearman}$ = 0.94, p < 0.001, n = 124975). However, the cumulative number of colonization events at the end of simulated time series was correlation with both the relative autocorrelation ($\rho_{spearman}$ = -0.49, p = 0.01) and the imbalance in rates ($\rho_{spearman}$ = 0.50, p = 0.01, n = 124975). Finally, the cumulative number of extinction events at the end of simulated time series was also correlated with the autocorrelation ($\rho_{spearman}$ = -0.54, p = 0.006) but not with the imbalance ($\rho_{spearman}$ = -0.29, p = 0.2, n = 124975).

Fig S5 – NICE over time across the different simulation settings. Dots represent a single simulation and the fitted line is from a linear mixed effect model having the simulation id as a random effect with the associated standard error. The first line (A to E) represents a strong imbalance in favor of colonizations, the second (F to J) a slight imbalance in favor of colonizations, the middle one (K to O) a perfect balance between colonization and extinction rates, the fourth (P to T) a slight imbalance in favor of extinction and the last one (U to Y) a strong imbalance in favor of extinction. The first column (A, F, K, P and U) represents a very low temporal autocorrelation, the second (B, G, L, Q and V) a low temporal autocorrelation, the middle one (C, H, M, R and W) the settings for observed autcorrelation (i.e., based on observed rates), the fourth (D, I, N, S and X) a high temporal autocorrelation and the last column (E, J, O, T and Y) a very strong temporal autocorrelation.

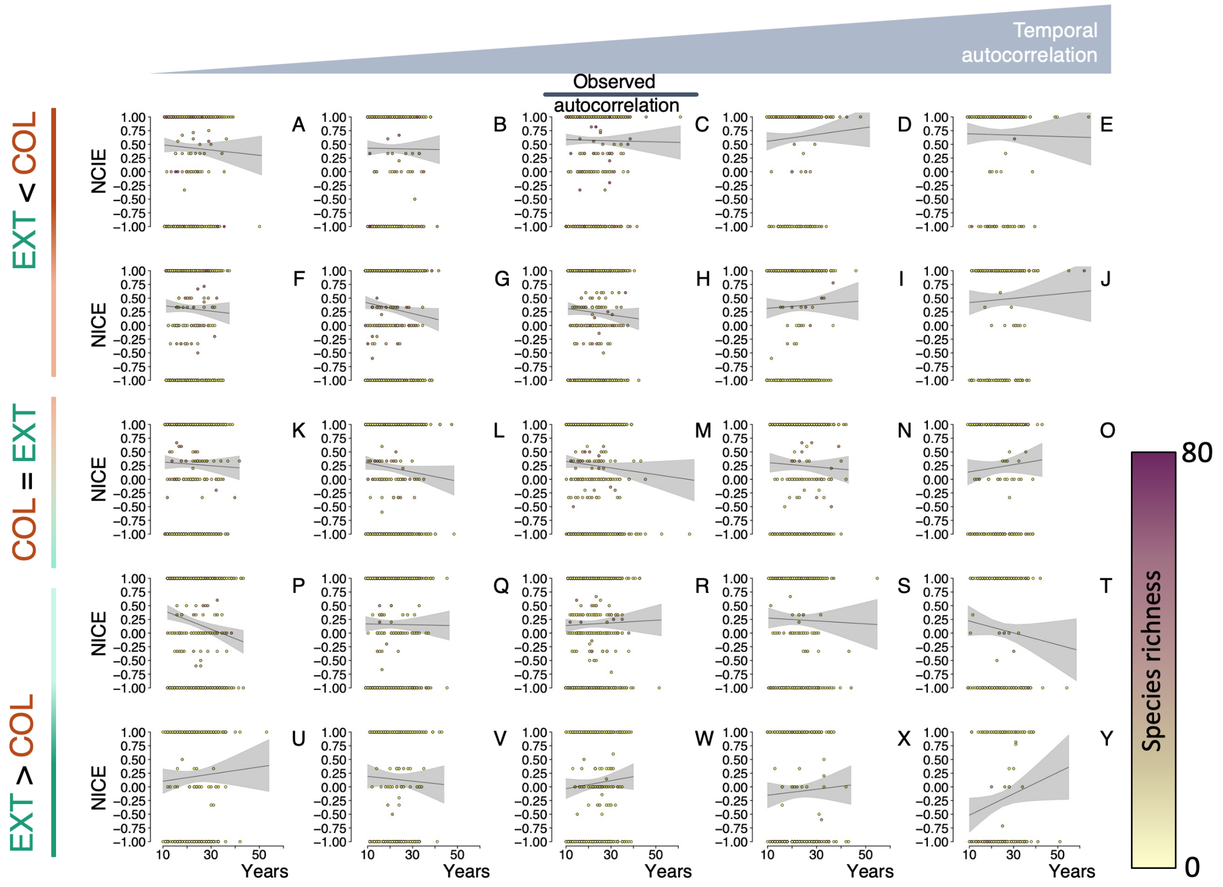

b. BBS settings

Final NICE values were not correlated with the relative autocorrelation ($\rho_{spearman}$ = -0.078, p = 0.7, n = 2810824) while being strongly indicative of the imbalance between colonization and extinction rates ($\rho_{spearman}$ = 0.98, p < 0.001, n = 2810824). Similarly, the cumulative number of colonization events at the end of simulated time series was not correlated with the relative autocorrelation ($\rho_{spearman}$ = 0.28, p = 0.2) but rather with the imbalance in rates ($\rho_{spearman}$ = 0.57, p = 0.003, n = 2810824). Finally, the cumulative number of extinction events at the end of simulated time series was also not correlated with the autocorrelation ($\rho_{spearman}$ = 0.11, p = 0.6) but with the imbalance ($\rho_{spearman}$ = -0.59, p = 0.002, n = 2810824).

Fig S6 – NICE over time across the different simulation settings. Dots represent a single simulation and the fitted line is from a linear mixed effect model having the simulation id as a random effect with the associated standard error. The first line (A to E) represents a strong imbalance in favor of colonizations, the second (F to J) a slight imbalance in favor of colonizations, the middle one (K to O) a perfect balance between colonization and extinction rates, the fourth (P to T) a slight imbalance in favor of extinction and the last one (U to Y) a strong imbalance in favor of extinction. The first column (A, F, K, P and U) represents a very low temporal autocorrelation, the second (B, G, L, Q and V) a low temporal autocorrelation, the middle one (C, H, M, R and W) the settings for observed autcorrelation (i.e., based on observed rates), the fourth (D, I, N, S and X) a high temporal autocorrelation and the last column (E, J, O, T and Y) a very strong temporal autocorrelation.

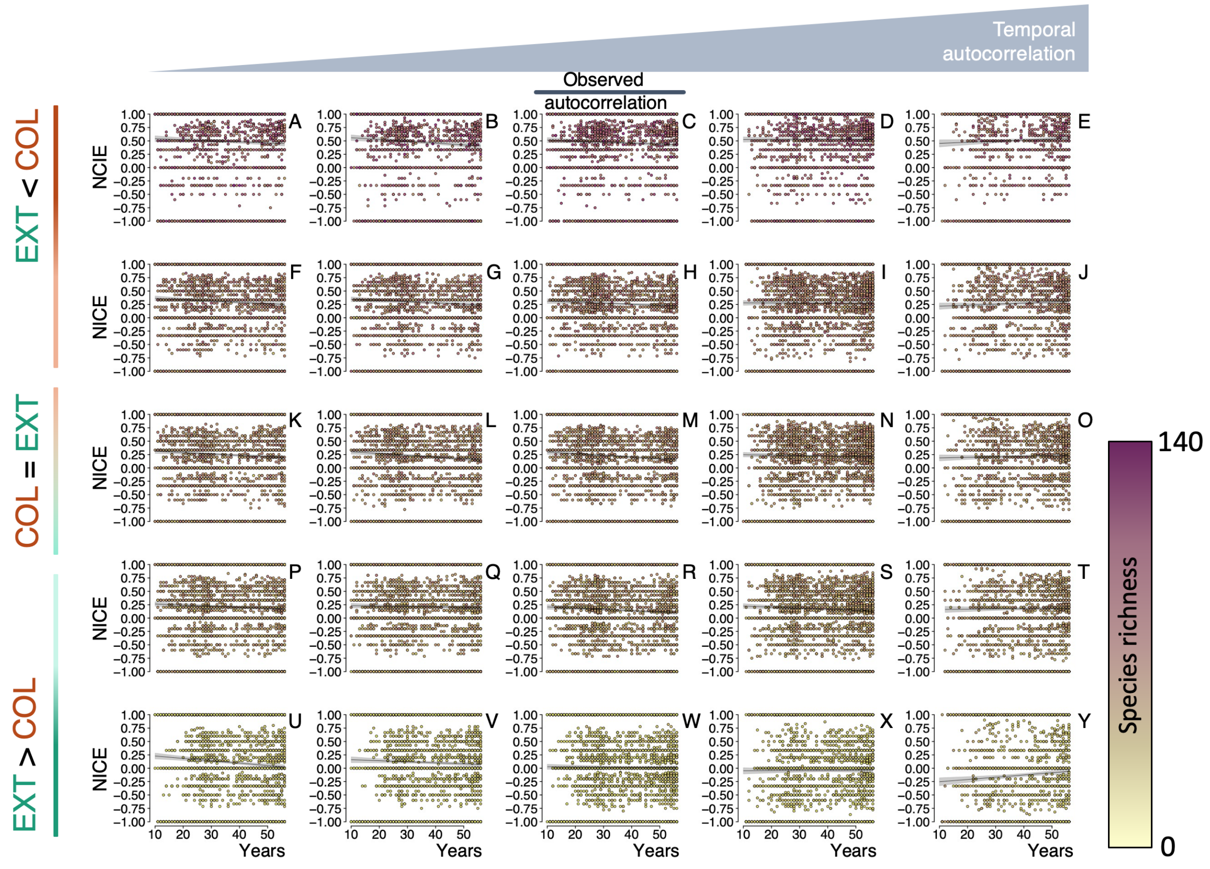

Supplement: Supplementary file 1 — Supplementary Figs. 1–6 and Tables 1–5. [file 41559_2023_2078_MOESM1_ESM.pdf]
